# Eponyms Describing Soft Tissue Pathologies in Orthopedic Hand Surgery

**DOI:** 10.7759/cureus.39449

**Published:** 2023-05-24

**Authors:** Jonathan Minto, Aman Andemichael, Thomas J Carroll, Danielle Wilbur

**Affiliations:** 1 Orthopedic Surgery, University of Rochester, Rochester, USA; 2 Orthopedic Surgery, Rutgers Robert Wood Johnson Medical School, New Brunswick, USA

**Keywords:** de quervain disease, dupuytren's contracture, eponyms, hand surgery, soft tissue

## Abstract

Orthopedic surgery literature utilizes numerous eponyms, and they have become commonplace among orthopedic surgeons and the general public alike. These eponyms can have important historical implications and their history is often overlooked by the physicians using such terms. This paper seeks to specifically explore the origins of eponyms in orthopedic soft tissue diseases involving the upper extremity. Shedding light onto the origin of these eponyms can provide greater respect and understanding of their use in orthopedic surgery today.

## Introduction and background

Modern orthopedic literature is filled with eponyms named after pioneers in the field. From fractures to diseases to diagnostic tests, eponyms are a path for surgeons to cement their legacy in the orthopedic vernacular. Despite the frequency of eponyms in orthopedics, modern curriculums do not stress the origins of these terms, thereby alienating the learner from orthopedic history. By studying the eponymous surgeons and scholars, and forming an understanding of the “why” or “how” something came to be, one is able to have a more complete picture with which to frame their understanding of orthopedic problems. This paper seeks to specifically explore the origins of eponyms in orthopedic soft tissue diseases.

## Review

Eponymous soft tissue diseases

De Quervain’s Tenosynovitis

De Quervain's tenosynovitis is a medical condition named after Fritz de Quervain, a Swiss physician born in Sion in 1868 [1]. After graduating from the University of Bern in 1892, he worked as the first assistant for the Nobel Prize-winning Dr. Theodor Kocher [1]. De Quervain eventually became the Chair of Surgery at the University of Basel in 1910 and then the head of the University Clinic of Surgery in Bern in 1918 [1].

De Quervain was the first physician to describe and treat chronic stenosing tenosynovitis at the radial styloid process [2]. He published several cases in an article titled "Ueber eine Form von chronischer Tenovaginitis" ("On a Form of Chronic Tendovaginitis") in 1895 [2]. In this article, he detailed both his observations and the surgical technique to correct this pain syndrome and made a note of differentiating it from the previously described "trigger finger" [2]. Today, De Quervain's tenosynovitis is defined as stenosing tenosynovial inflammation of the first dorsal compartment [2].

Dupuytren’s Contracture

Dupuytren’s contracture, also known as Dupuytren’s disease, is named after the French surgeon Baron Guillaume Dupuytren [3]. He first operated on the disease in 1831 and published his findings in *The Lancet* in 1834 [3]. Dupuytren was born in 1777 in Pierre-Buffière, France, and received his doctorate in 1803 [4]. He began working at the Hôtel-Dieu in Paris shortly after [4]. In 1812, he was appointed professor of operative medicine at the Faculty of Medicine of Paris and later became president of the Academy of Medicine in 1824 [4]. He was even chosen as the personal surgeon to Louis XVIII and Charles X [4].

There has been historical debate about whether the eponym should be named after Dupuytren, as two surgeons proposed treatments for the palmar contractures before him (Henry Cline Sr. and Astley Cooper) [4]. The terms “Cline’s contracture” and “Cooper’s contracture” are still used to describe the disease [4].

Dupuytren’s disease is characterized by an abnormal thickening of the fascia, which can develop into a thick cord that prevents one or more digits from contracting [3]. The fourth and fifth digits are most commonly affected, resulting in the classic “Hand of Benediction” sign, inspired by an early Pope who suffered from Dupuytren's disease [3].

*Volkmann* *Contracture*

Richard von Volkmann, born in Leipzig, Germany in 1830, was a professor of surgery at the University of Halle from 1867 [5]. He attended medical school in Giessen, Halle, and Berlin, from which he graduated in 1854 [5]. In 1864, he wrote a chapter on "Dysfunctions of the musculoskeletal system" for the *Handbook of Basic and Special Surgery*, where he first described the contracture that now bears his name [5]. He reiterated his findings in 1881, stating that he believed “that the pareses and contractures of limbs following application of tight bandages are caused not by pressure paralysis of nerves, as formerly assumed, but by the rapid and massive deterioration of contractile substance and by…reactive and regenerative processes” [6].

Today, Volkmann's contracture, also known as Volkmann's ischemic contracture, is defined as a permanent flexion contracture of the hand at the wrist, resulting in a claw-like deformity of the hand and fingers [5].

Apert Syndrome

Apert syndrome is named after French pediatrician Eugène Charles Apert. This is one of the several congenital abnormalities with parallels in the animal kingdom [7]. Born in 1868 in Paris, Apert received his doctorate in 1897 and later worked at the Hôtel-Dieu and Hôpital Saint-Louis from 1919 until 1934 [7]. Apert's interest in the upper limb structure of higher-order animals led him to observe the resemblance between the paddle of a turtle and a human hand with acrosymbrachydactyly [7]. In addition to joined fingers, Apert syndrome (also called acrocephalosyndactyly) involves craniosynostosis and maxillary hypoplasia [7].

Wartenberg’s Sign and Syndrome

Robert Wartenberg, a neurologist, was born in Grodno, old Lithuania (present-day Belarus), in 1887 [8]. He received his medical degree from the University of Rostock in 1919 and worked at the University of Freiburg until 1935 when he immigrated to the United States to escape Nazi Germany [8]. He worked at the University of California, San Francisco, and became a Clinical Professor of Neurology in 1952, writing over 150 papers on clinical neurology in German and English, with a focus on reflexes and signs [8].

In 1932, Wartenberg described five cases of mononeuropathy of the superficial branch of the radial nerve, which he named "cheiralgia paresthetica" or hand pain [9]. Superficial radial nerve palsy, superficial radial nerve compression, and cheiralgia paresthetica are synonymous with what is now often called Wartenberg’s syndrome [9]. This condition is characterized by pain, paresthesia, and dysesthesia along the dorsoradial distal forearm extending onto the dorsal first web space and thumb, with symptoms often worsening with wrist movement [9].

In addition to the eponymous syndrome, Wartenberg’s name is affixed to a sign of ulnar nerve palsy [9]. This sign, as described by Wartenberg, “consists of a position of the abduction ASSUMED by the little finger” [9]. If the ulnar nerve is injured, intact radial nerve function can result in unopposed abduction of the little finger [9].

Vaughan-Jackson Syndrome

Vaughan-Jackson syndrome is a specific pathophysiologic process that leads to extensor tendon rupture in a rheumatoid wrist. Vaughn-Jackson syndrome was originally defined in 1948 by Dr. OJ Vaughan-Jackson, who described a unique set of two patients that had extensor tendon rupture due to attrition at an arthritic distal radio-ulnar joint [10]. He described a pathologic process where an arthritic roughening of the articular margin of the distal end of the ulna erodes through the joint capsule of the wrist, directly contacting the extensor tendon to the fifth digit leading to tendon rupture [11].

Patients with Vaughan-Jackson syndrome typically experience a sudden loss of finger extension starting at the fifth digit and progressing radially toward the second digit [11]. Therefore, some patients may present with multiple extensor tendon ruptures and not just an isolated rupture of the fifth extensor tendon [11]. Treatment involves surgery with a combination of tenosynovectomy, tendon transfers, and distal ulna resection or arthroplasty [11].

## Conclusions

Eponyms are often a frequent source of frustration for the orthopedic learner, providing little connection to the underlying disease, or evaluation to which they belong. Yet, they provide an opportunity to understand the greater context of orthopedics and the pioneers who came before us. Connecting with this history allows practitioners to learn from the past to develop a greater understanding of present problems. Regardless of the degree to which eponyms persist in the orthopedic vernacular or education, what ought to persist is the history the eponym carries with it.

## References

[1] Ahuja NK, Chung KC (2004). Fritz de Quervain, MD (1868-1940): stenosing tendovaginitis at the radial styloid process. J Hand Surg Am.

[2] Caldwell RA, Shorten PL, Morrell NT (2019). Common upper extremity fracture eponyms: a look into what they really mean. J Hand Surg Am.

[3] Zdilla MJ (2017). The hand of Sabazios: evidence of Dupuytren's disease in antiquity and the origin of the hand of Benediction. J Hand Surg Asian Pac Vol.

[4] Gudmundsson KG, Jónsson T, Arngrímsson R (2003). Guillaume Dupuytren and finger contractures. Lancet.

[5] Somford MP, Wiegerinck JI, Hoornenborg D, van den Bekerom MP, Eygendaal D (2015). Eponyms in elbow fracture surgery. J Shoulder Elbow Surg.

[6] Willy C, Schneider P, Engelhardt M, Hargens AR, Mubarak SJ (2008). Richard von Volkmann: surgeon and Renaissance man. Clin Orthop Relat Res.

[7] Thurston A (2011). Vital endowments: Sir Charles Bell and the history of some congenital abnormalities of the upper limb. ANZ J Surg.

[8] Burkholder DB, Boes CJ (2021). Robert Wartenberg and the American Academy of Neurology. Neurology.

[9] Kuschner SH, Berihun H (2021). Robert Wartenberg syndrome and sign: a review article. Open Orthop J.

[10] Vaughan-Jackson OJ (1948). Rupture of extensor tendons by attrition at the inferior radio-ulnar joint; report of two cases. J Bone Joint Surg Br.

[11] Sanderson CJ, Kaye MB (2020). Vaughn-Jackson syndrome. Open Access J Intern Med.

